# Efficacy and Dose-Dependent Safety of Intra-Arterial Delivery of Mesenchymal Stem Cells in a Rodent Stroke Model

**DOI:** 10.1371/journal.pone.0093735

**Published:** 2014-05-07

**Authors:** Dileep R. Yavagal, Baowan Lin, Ami P. Raval, Philip S. Garza, Chuanhui Dong, Weizhao Zhao, Erika B. Rangel, Ian McNiece, Tatjana Rundek, Ralph L. Sacco, Miguel Perez-Pinzon, Joshua M. Hare

**Affiliations:** 1 Cerebral Vascular Disease Research Laboratories, Leonard M. Miller School of Medicine, University of Miami, Miami, Florida, United States of America; 2 Department of Neurology, Leonard M. Miller School of Medicine, University of Miami, Miami, Florida, United States of America; 3 Interdisciplinary Stem Cell Institute, Leonard M. Miller School of Medicine, University of Miami, Miami, Florida, United States of America; 4 The Department of Medicine, Leonard M. Miller School of Medicine, University of Miami, Miami, Florida, United States of America; Tokai University, Japan

## Abstract

Intra-arterial (IA) delivery of mesenchymal stem cells (MSCs) for acute ischemic stroke is attractive for clinical translation. However, studies using rat model of stroke have demonstrated that IA MSCs delivery can decrease middle cerebral artery (MCA) flow, which may limit its clinical translation. The goal of this study is to identify a dose of IA MSCs (maximum tolerated dose; MTD) that does not compromise MCA flow and evaluate its efficacy and optimal timing in a rat model of reversible middle cerebral artery occlusion (rMCAo). We sought to determine if there is a difference in efficacy of acute (1 h) versus sub-acute (24 h) IA MSCs treatment after rMCAo. Adult female Sprague-Dawley rats underwent rMCAo (90 min) and an hour later a single dose of MSCs (at de-escalating doses 1×10^6^, 5×10^5^, 2×10^5^, 1×10^5^ and 5×10^4^) was given using IA route. MSCs were suspended in phosphate buffered saline (PBS) and PBS alone was used for control experiments. We measured the percent change in mean laser Doppler flow signal over the ipsilateral MCA in de-escalating doses groups to determine MTD. The results demonstrated that the lowering of IA MSC dose to 1×10^5^ and below did not compromise MCA flow and hence an IA MSC dose of 1×10^5^ considered as MTD. Subsequently, 1 h and 24 h after rMCAo, rats were treated with IA MSCs or PBS. The 24 h delivery of IA MSCs significantly improved neurodeficit score and reduced the mean infarct volume at one month as compared to control, but not the 1 h delivery. Overall, this study suggests that the IA delivery of MSCs can be performed safely and efficaciously at the MTD of 1×10^5^ delivered at 24 hours in rodent model of stroke.

## Introduction

Stroke is the main cause of long-term disability and the third leading cause of death in the United States. The public health burden of stroke continues to be staggering with an estimated cost of 73.3 billion in 2010 [1]. Despite the approval of intravenous recombinant tissue plasminogen activator (rtPA) 18 years ago, and rapid growth in number of endovascular recanalization therapies for acute ischemic stroke (AIS), their impact on reducing stroke-related long-term disability is limited [2], [3]. Hence, there continues to be a critical need for novel therapies for AIS. In this regard, over the last decade, various types of stem cell have been tested in several pre-clinical studies suggesting improved functional neurological outcomes after AIS [4]–[7]. The leading type of stem cell for clinical translation in stroke is the mesenchymal stem cell, which is an adult, non-hematopoietic progenitor cell with capacity to differentiate into a variety of cell lineages including osteoblasts, chondrocytes and neuron-like cells [8]–[11].

Mesenchymal stem cells (MSCs) are multipotent non-hematopoietic stem cells found mainly in the stromal fraction of the bone marrow as well as in the connective tissue of most organs [9]–[11]. MSCs can be easily isolated from adipose tissue, amniotic fluid, placenta and umbilical cord, though they are most commonly and efficiently derived from adult bone marrow. MSCs are an attractive cell source because they are relatively easy to obtain, expand and manipulate *in vitro* [12]. Moreover, adult MSCs do not confer the risk of tumorgenicity that pluripotent cells carry [13]. Importantly, MSCs are also immunoprivileged, with low MHC I and no MHC II antigen expression [12], [13]. Their immunoprivileged nature obviates the need for immunosuppression in allogeneic administration of MSCs. Thus allogeneic MSCs, already produced from a healthy donor, can be given as an off-the-shelf product without delay if needed without the need of immunosuppression. This feature is especially attractive for future translation of MSCs into a treatment for ischemic stroke, which is presents most often without warning and may benefit from early administration of cell therapy [14]–[16].

There are multiple routes of stem cell delivery to the brain in AIS. Among these, the intra-arterial (IA) route of stem cell delivery has a high potential for clinical translation, in view of the increasing clinical application of endovascular therapy in the treatment of AIS [6], [17]–[20]. Furthermore, IA delivery of stem cells after AIS is minimally invasive and results in a larger number and more diffuse distribution of stem cells in and around the infarcted area when compared to intra-parenchymal, intra-cerebroventricular and intravenous stem cell delivery [21]. IA delivery avoids the first pass trapping of stem cells in the lungs and liver seen with intravenous delivery [22]. Also, a recent study has shown superior functional and histological outcomes with IA delivery of stem cells as compared to intravenous administration [23]. However MSCs range in size from 5–50 microns and a major limitation to IA delivery of MSCs is the possibility of compromise of regional cerebral blood flow (rCBF) owing to those MSCs in the larger size range of 20–50 microns; thus a potentially novel therapeutic strategy can paradoxically worsen of cerebral ischemia outcome [10].

In the current study, first we hypothesized that compromise of MCA blood flow during IA delivery of MSCs was dose-dependent and therefore, lowering the dose of IA MSCs would mitigate rCBF compromise. The efficacy of IA MSC in focal cerebral ischemia, with doses lower than 1×10^6^ has not been studied. Hence, we also hypothesized that the lower safe dose we identify, based on our first hypothesis, would be efficacious in focal cerebral ischemia. Furthermore, the efficacy of IA MSCs given immediately after reperfusion (within 60 minutes) as compared to the efficacy when given at 24-hours is unknown. Hence, we additionally hypothesized that the efficacy of the MTD of IA MSCs delivered at 24 hours (sub-acute) and 60 minutes (acute) in a rodent cerebral ischemia model would be the same.

## Materials and Methods

All animal procedures were carried out in accordance with the Guide for the Care and Use of Laboratory Animals published by the U.S. National Institutes of Health and approved by the Animal Care and Use Committee of the University of Miami.

Female Sprague-Dawley rats (Charles River Laboratories, Inc., Wilmington, MA) weighing 260–310 g were used. The animals in the experimental groups were allocated in a randomized fashion. Investigators were blinded to dose and treatment group allocation during surgery and during outcome evaluations.

### Surgery

The animals underwent Reversible Middle cerebral artery occlusion (rMCAo) following an overnight fast. Anesthesia was induced with 3% isofluorane and 70% nitrous oxide. Rats were intubated endotracheally and ventilated mechanically on a mixture of 1–0.5% isofluorane, 70% nitrous oxide and a balance of oxygen. The right femoral artery and vein were catheterized to permit to monitor blood pressure and to take arterial samples for blood gas and glucose assessments. Arterial *P*CO_2_ and *P*O_2_ were maintained in the normal range by ventilator adjustment. For immobilization, rats received pancuromium bromide 0.75 mg/kg i.v. Rectal temperature was measured continuously and maintained at 37–37.5°C by a heating pat under the rat's body. Cranial temperature was separately monitored by a 29-gauge thermocouple implanted into the right temporalis muscle and was maintained at 36–36.5°C by a warming lamp placed above the rat's head throughout the experiment. Our previous studies have shown that the cranial temperature 36–36.5°C corresponds to a brain temperature of 36.5–37°C [24]. Physiological variables (plasma glucose concentration, pH, PCO_2_, PO_2_ and mean arterial blood pressure (MABP)) were maintained normal before and after ischemia (Table S1 and S2; presented as supplementary data).

### CBF monitoring

The left scalp was opened and the skull was exposed. A 2-mm bur hole was drilled on the left *sphenoid*, 3 mm below the up edge, 0.5 mm anterior and 6 mm lateral to the bregma, leaving the dura intact. A short Laser Doppler Flowmetry (LDF, Perimed Inc.) probe was placed above the dura and fixed on to the skull with glue and dental cement. From this point, the CBF inside the trunk of the ascending vessel from cortical branch of MCA was monitored. The LDF signal reading was recorded, starting at 30 min before the suture insertion and continuously throughout the experiment and 60 min after the cell infusion, using a specific designed monitoring system.

### Reversible Middle cerebral artery occlusion (rMCAo)

The previously well-described suture technique for MCAo was utilized in this study [25]–[28]. The common carotid artery (CCA) was exposed through a midline incision on the ventral neck and carefully separated from the surrounding tissue including the adjacent vagus nerve by blunt dissection using microsurgery. Under an operation microscope, the branches of external carotid artery (ECA), superior thyroid and ascending pharyngeal arteries and further the terminal lingual and maxillary artery branches, were dissected and coagulated. Two long 5–0 silk sutures tied the ECA as distal to carotid bifurcation as possible. Two short (3 cm) 5–0 silk sutures were tied loosely around the segment of ECA closed to the bifurcation. The ICA was exposed, and the origin of the pterygopalatine artery (PPA) was visualized. An intraluminal 3.0 nylon suture, having a spherical and enlarged tip produced by heating near flame, introduced into the distal segment of ECA via a small incision on the vessel and advanced into the ECA lumen to reach the bifurcation. The 2 short silk sutures subsequently were tied to prevent bleeding. The ECA was cut at the distal segment between the 2 long-suture notes. The stump of the ECA was held toward the surgeon; thus the stump and the ICA were on a straight line. The suture was then turned into ICA with a special care not enter the PPA. The suture went advance in ICA until the sharp drop of regional cerebral blood flow confirmed by the LDF. The length of inserted suture inside the ICA from the bifurcation was 19–25 mm. A sudden and sharp decrease in the LDF signal was interpreted to indicate a successful MCA occlusion. The suture was gently withdrawn after the 90-min occlusion period. During the procedure and the 2-h recirculation, blood pressure and the blood gas were maintained in the normal ranges, cranial and rectal temperature were maintained at normal levels.

### Inclusion and Exclusion Criteria

Rats that had less than 50% drop in LDF signal on suture insertion and premature mortality during rMCAo were excluded from the study prior to randomization and allocation to treatment groups.

## Experimental Design

### (1) Safety Study

**Figure 1:**
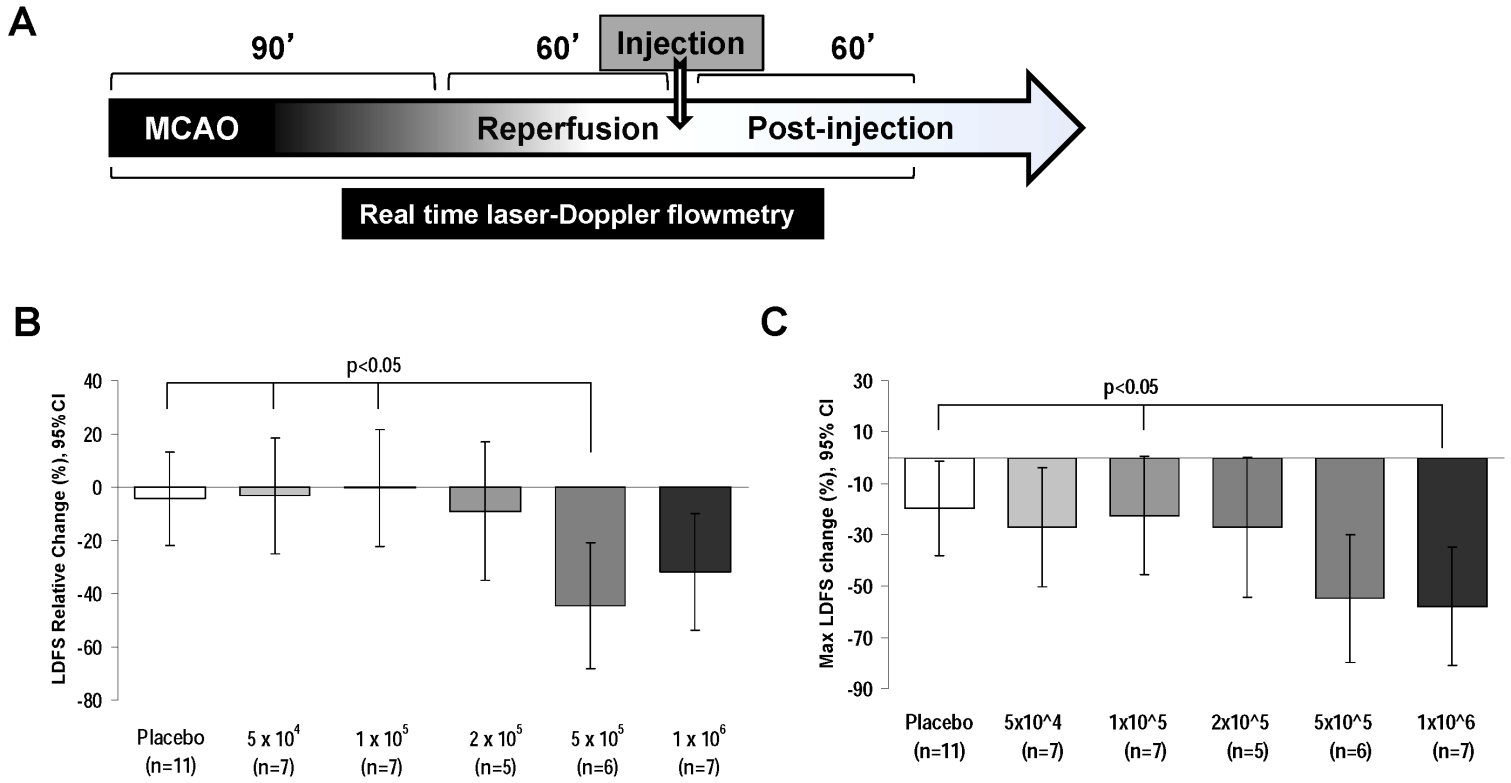
Lower doses of IA MSCs mitigate adverse effect IA injection on MCA blood flow. (A) Experimental timeline showing rMCAo for 90 minutes followed by withdrawal of the suture to allow reperfusion. At 60 minutes of reperfusion, IA MSC or vehicle only injection was given, followed by LDF monitoring for 60 min. (B) Comparison of relative LDFS worsening from baseline to final recording, among de-escalating dose groups (C) The 1×10^5^ dose and placebo has significantly less maximum LDFS worsening as compared to the 1×10^6^ dose. The comparisons in B and C were done using general linear modeling (GLM) to compare mean differences among groups. LDFS =  Laser Doppler Flow Signal.

Forty-three female rats underwent rMCAo. The suture was gently withdrawn after a 90-minute occlusion period (Figure 1A, B). At 60 minutes after recirculation, a 1 ml syringe was filled with the MSCs suspended in phosphate buffered saline (PBS) or PBS alone. The cell suspension in 0.5 mL PBS or PBS alone (0.5 mL) was delivered into the ipsilateral IA over 3 minutes. The different groups of animals received 1×10^6^, 5×10^5^, 2×10^5^, 1×10^5^ & 5×10^4^ MSCs in PBS or only PBS. Following MSCs or PBS delivery, the catheter was carefully withdrawn and the ECA was tied. During the surgical procedure of rMCAo and IA MSCs deliver, we measure blood flow over ipsilateral MCA using laser Doppler flow signal (LDFS).

### (2) Efficacy Study

In another cohort, 34 female rats underwent 90 minutes of rMCAo. Twenty-four hours after induction of rMCAo, rats were assigned to receive IA MSCs (1×10^5^) or IA PBS or intravenous (IV) MSCs 1×10^6^. In the IV MSCs group, the cells were given into the femoral vein at a 10-fold of the IA MSC dose in order to potentially increase the number of MSCs reaching the injured brain. In an additional experimental group, IA MSCs at 1×10^5^ were given at 60 minutes after rMCAo to study the effect of acute timing on efficacy. The primary outcome measure was neurodeficit score and the secondary outcome was infarct volume at 4 weeks.

### Donor rodent MSCs

MSCs used in this study were derived from green fluorescence protein (GFP) transgenic male Sprague-Dawley rats. At the Cell Manufacturing Program at the University of Miami, the bone marrow was harvested from rats and the mononuclear cells isolated by density gradient centrifugation. The cells were then cultured at 1 to 5×10^6^ cells per ml in 25 ml of alpha MEM plus 20%FCS in T162 cm^2^ culture flasks at 5%CO_2_ and 37°C. MSCs exhibited spindle-shaped morphology and were characterized by (i) adherence to plastic, (ii) negativity for hematopoietic cell surface markers CD34 and CD45 (0.1±0.1% and 0.2±.0.1%, respectively) and positivity for CD73, CD90.2, and CD105 (88±5.4%, 99.2±0.7% and 95±3.7%, respectively). The cells were expanded to passage 6 and then the MSCs were suspended in 10% of cryopreservant dimethylsulfoxide (DMSO) and frozen in liquid nitrogen. Prior to injection, the cells were thawed rapidly and washed to remove (DMSO), and then re-suspended in PBS to the required cell dose. We evaluated cell viability prior to infusion using trypan blue technique. Only cell doses at and above 70% viability were administered in the study.

The presence of hematopoietic cell surface markers was investigated using fluorescence-activated cell sorting (FACS). Briefly, a total of 0.5−1×10^6^ MSCs was used for FACS characterization. GFP positivity was detected in 79±5% of MSCs in culture. For surface markers, cells were incubated for 1 hour with FACS buffer (1% bovine serum albumin and 5% FBS diluted in distilled water) on ice, and subsequently 1 hour with the primary and secondary antibodies (washed 3 times for 5 minutes during centrifugation between the primary and secondary and after the secondary). Each analysis included at least 10,000 events and was performed on at least three separate cell preparations (BD FACSAria, University of Miami). All experiments for MSCs characterization were performed between passages 8 to 11 after isolation. Cells were incubated with anti-mouse PE-fluorochrome-conjugated antibodies against CD45, CD73, and CD90.2 (BD Biosciences, San Jose, CA), PE-fluorochrome-conjugated against CD34 (Santa Cruz Biotechnology, Santa Cruz, CA), PE/Cy7-fluorochrome-conjugated against CD105 (BioLegend, San Diego, CA), and their respective isotype controls (BD Biosciences, San Jose, CA).

### Intra-arterial MCA infusion

A PE-10 polyethylene catheter connected to a 30G needle and a 1-ml syringe was inserted into the stump of left ECA through an incision on the vessel under the operation microscope and was tightened to ECA by a 5–0 silk suture. The catheter went forward into ICA and avoided PPA, the extracranial branch of ICA, to approach the skull base [29]. The catheter was filled with heparinized physiological saline to prevent air bubble and coagulation. The syringe was filled with MSCs in PBS or PBS only. Using slow hand injection over 3 minutes, 0.5 ml volume of the cells in PBS or PBS only were then delivered into the ICA via the PE-10 catheter.

### Histology and detection of MSCs in the brain

Under anesthesia rats were perfused via ascending aorta with FAM (a mixture of 40% formaldehyde, glacial acetic acid, and methanol, 1∶1∶8 by volume) for 20 min following a 2-min initial perfusion with physical saline. The rat heads were immersed in FAM for 1 day before the brains were removed. The brains were placed in FAM at 4°C for at least 1 additional day, and then coronal brain blocks were embedded in paraffin. All brains were cut into 10- µm thick sections from 5.5 mm to −7.5 mm from bregma at 9 standard levels to cover the whole infracted areas. Sections of the 9 levels were stained with hematoxylin and eosin to display the infracted areas and to obtain infarct volumes.

In a parallel study, adjacent sections were subjected to immunohistochemistry (IHC) studies with Vectastain ABC peroxidase method (Vector Labs, Burlingame, CA). Sections from levels 2–8 in the dose group 1×10^5^ & 5×10^5^ were tested by GFP-IHC to reveal the MSCs immigration inside the brain via injection through IA. To detect GFP positive MSCs, sections were incubated in the working dilution of 1∶100 of anti-GFP (SC-101525, Santa Cruz Biotech, CA.) for 90 min at room temperature, and 3, 3″-diaminobenzidine (DAB) was used for visualization of the primary antibody binding. The dark-brown appearance was considered the specific labeling to indicate the MSCs. Hematoxylin counterstain was conducted to identify cells and brain regions. Blocked vessels were defined as: complete filling of the microvessel caliber with MSCs in axial section or segment of vessel in sagittal section of a vessel. The absolute total number of blocked microvessels per section was reported.

Both positive control, to confirm the negative labeling being truly negative, and negative control, to confirm the positive staining not false positive, were applied in the present study. The two negative controls including 1) mouse IgG 1 (X0931, Dako, Carpenteria, CA) replaced anti-GFP in the female brains of the present study and 2) anti-GFP applied to male rat brains having infarction from previous study were performed. Negative labeling represented in the 2 negative controls supported that the positive staining were true positive. Anti-glial fibrillary acidic protein (GFAP, Dako, Carpenteria) was employed in the IHC study as the positive control to ensure that the reduced sensitivity of reaction not due to improper technique because positive control slide, i.e. the known histological slide containing the specific antigen unavailable [26].

### Neurodeficit Scoring

A standardized neurobehavioral test battery was conducted as described previously [27], which includes tests for postural reflex, sensorimotor integration and proprioception. Total neurological score ranged from a normal score of 0 to a maximal possible score of 12.

### Statistical analyses

Descriptive statistics for physiological continuous variables were presented as means with standard deviations. For safety analysis, the primary outcome was the relative change in MCA blood flow from pre-injection to the final assessment after injection of MSCs or saline, whereas the secondary outcome was the maximum relative reduction in blood flow within the 60 minutes after injection. General linear model (GLM) was used to compare mean differences among groups in the relative change at the final LDFS measurement and in the maximum relative reduction in blood flow. Mixed model was used for analyzing for comparing LDFS over time between two groups. For efficacy analysis, mixed-effects model was used to assess differences among groups in neurodeficit scores based on the repeated measures. Due to skewed distribution, log transformation was first performed for infarct volume and ANOVA was used to compare the differences among groups at week 4 after treatment. All analyses were performed with SAS 9.3 (SAS Institute Inc., Cary, NC) or SPSS 16.0 (SPSS Inc., Chicago, IL).

## Results

### Lower IA MSC doses mitigate adverse impact on MCA blood flow

**Figure 2:**
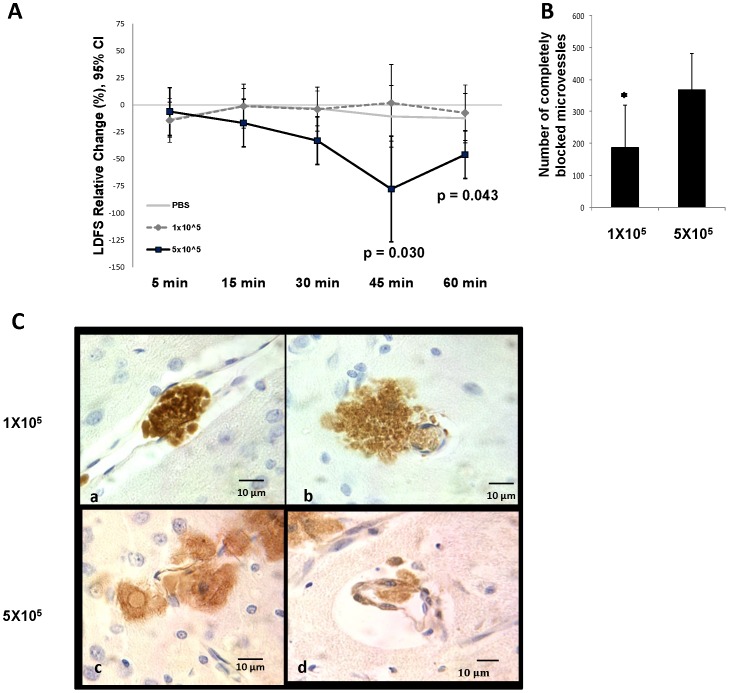
Comparison of LDFS changes over time and microvascular occlusion between dose-groups. (A) IA MSC dose of 1×10^5^ and IA placebo have a transient and less MCA LDF worsening during 60 minutes post injection as compared to 5×10^5^ dose using mixed model. LDFS =  Laser Doppler Flow Signal. (B) On comparing the total number of microvessels with complete occlusion among the two dose groups, there were a significantly lower number of complete occlusions in the lower dose group. Mean ±SD, * P<0.05, ANOVA. (C) Representative brain sections from IA MSC 1×10^5^ dose group showing GFP+ MSCs identified by 3, 3″-diaminobenzidine (DAB), showing localized complete filling of microvessels at 3–5 days post-injection, as well as MSCs just outside the vessel wall in brain parenchyma and (D) high power field of representative brain sections from IA 5×10^5^ dose group showing single MSC partly inside and partly outside microvessel wall.

We first determined the impact of lowering the dose of IA MSC on MCA blood flow, starting with a dose of 1 million cells, which was previously shown to severely decrease MCA flow in one-thirds of animals [10]. In the animals receiving 1×10^6^and 5×10^5^ MSCs, there was a considerable decrease in the final post-injection MCA flow by 32% and 45% respectively, compared to the pre-IA MSC injection MCA flow (Figure 1B). Lowering the dose below 5×10^5^ resulted in mitigation of the adverse effect of IA MSC injection on MCA flow. At the dose of 2×10^5^, the MCA flow compromise was less as compared to the 5×10^5^ dose group, but this was non-significant (LDFS decreased by 9% vs 45%, p = 0.06). At the next lowered dose level of 1×10^5^ MSCs, the MCA flow compromise was significantly less as compared to 5×10^5^ dose group (LDFS decreased by 0.2% vs 45%, p = 0.01). When we analyzed the maximum reduction in LDFS among the dose groups, the rats receiving 1×10^6^and 5×10^5^ MSCs had a maximum reduction of LDFS over 50%, whereas those receiving a dose at 1×10^5^ MSCs had a maximum reduction in LDFS of 23%, similar to 20% in placebo group (Figure 1C). Furthermore, we found that as compared to the 5×10^5^ dose, the 1×10^5^ IA MSC dose injection led to less worsening in LDFS post injection over the course of 60 minutes post injection, becoming significantly improved at 45 minutes and 60 minutes post-injection (Figure 2A). Based on these results the 1×10^5^ IA MSCs dose was utilized for subsequent studies and defined as maximum tolerated dose (MTD).

### Mortality with MCAo and IA MSCs

The mortality rate of the MCAo procedure (premature mortality) in this study was 13.46%. In the part of the study evaluating safety of de-escalating doses, the mortality rate after IA MSC dose administration was: 8.3%, 28.6%, 12.5%, 42.9%, 42.9% and 42.9% in the placebo, 5×10^4^, 1×10^5^, 2×10^5^, 5×10^5^ and 1×10^6^ IA MSC dose groups respectively.

### Impact of MSC Dose on Microvascular Occlusion and MSC Distribution

Consistent with the effect on MCA flow, at 3–5 days post injection, we found a significantly higher number of brain microvessels showing complete occlusion with MSCs in rats receiving IA MSCs at the higher dose of 5×10^5^ as compared to the MTD of 1×10^5^(366.5 vs. 185.75, p = 0.047) (Figure 2B). However, in rats in both dose groups, MSCs were also seen in the brain parenchyma just outside the microvessels at the level of the blockage. (Fig. 2C) We noted two sections in one rat from the larger dose group in which individual MSCs were located across the vessel wall partly inside the vessel and rest in the adjacent brain parenchyma, consistent with them being in the process of diapedesis.

### Efficacy Study of MTD of IA MSCs

**Figure 3:**
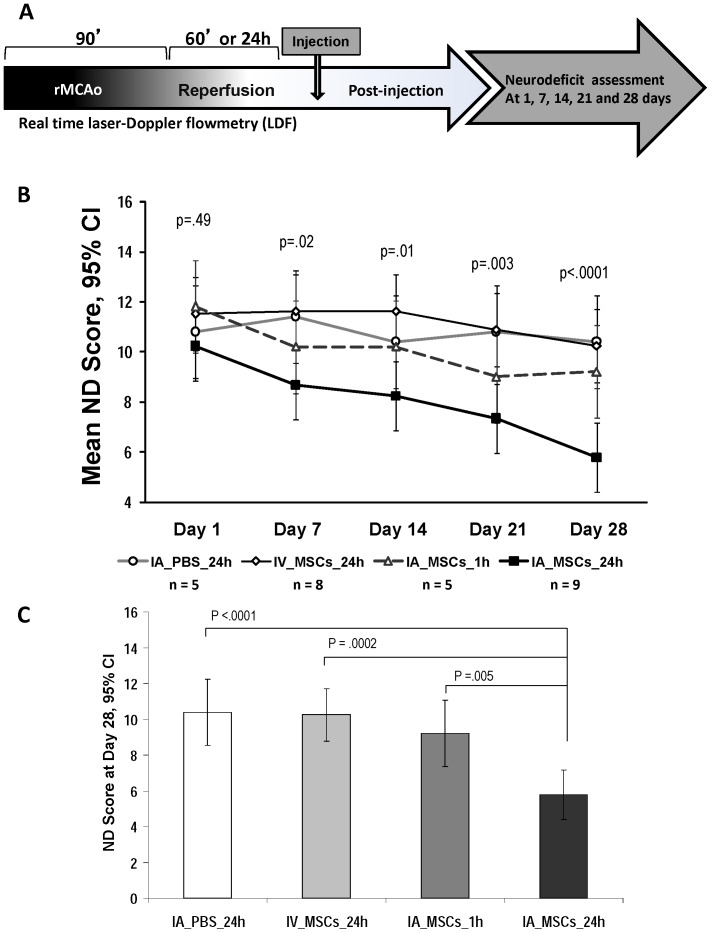
Functional neurologic outcomes are superior in the IA MSC_24 h group. (A) Experimental timeline of the efficacy study showing 90 min rMCAo followed by 24 hour reperfusion except in group receiving IA MSCs at 1 hour reperfusion followed by ND score assessment at 1,7,14,21 & 28 days. (B) On day 1 post rMCAo, the ND scores were not significantly different among groups. The ND score of the 1×10^5^ group progressively decreased over time and at 28 days was significantly lower than the other groups. C, The day 28 ND score was significantly lower in the IA MSC_24 h group as compared to all the remaining groups. ND =  Neurodeficit

We chose the 1×10^5^ MSCs as the dose to use for our efficacy study of IA MSCs in rMCAo based on the above results showing that this was the highest dose group that had minimal adverse effect on MCA LDF post-injection. We compared the efficacy of IA MSCs given at 24 hours (IA MSC_24 h), at one hour (IA MSC_1 h), IV MSCs at a ten-fold dose given at 24 hours (IV MSC_24 h) and IC PBS given at 24 hours (IA PBS_24 h) post rMCAo. On mixed model analysis we found a significant interaction between treatment group and time point (p = 0.02), with no significant difference among the groups at day 1 (p = 0.49) and most significant difference at 28 days (p<0.0001) after treatment (Figure 3B). The IA MSC_24 h group had a significantly lower neurodeficit score (5.8, 95% CI: 4.4 to 7.2) at 28 days after treatment as compared to IA PBS_24 h (10.4, 8.5 to 12.3, p<0.0001), as well as to IV MSC_24 h at dose of 1×10^6^ (10.3, 8.8 to 11.7, p = 0.0002), and IA MSC_1 h (9.2, 7.3 to 11.1, p = 0.005) groups. The IA MSC_1 h group showed no significant difference in functional outcome compared to the IA PBS_24 h (p = 0.37) and IV MSC_24 h (p = 0.38) groups (Figure 3C).

**Figure 4:**
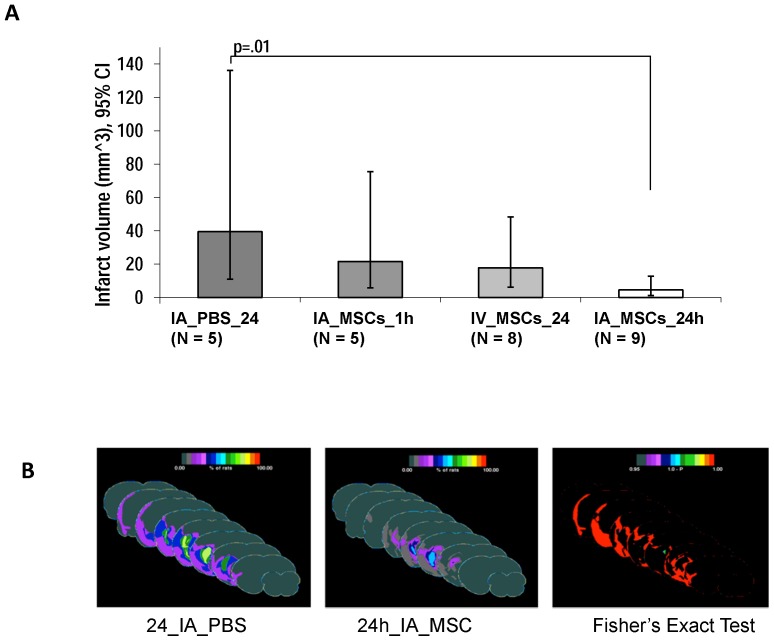
Comparison of infarct volume among treatment groups. (A) Geometric mean infarct volumes are compared among groups after log transformation to achieve normal distribution. Only the IA MSC_24 h group shows significantly reduced infarct volume as compared to the IA PBS_24 h. B, Frequency infarct map statistically comparing the location of the mean infarct volume in the IA MSC_24 h and IA PBS_24 h groups using color- coded representation of the percent of rats showing infarction in each brain region using “Fisher's exact test” with a color-coded representation of the “p-value”, the color bar in “1-p” format. Displays are in “Coronal presentation”; middle sections are selected. The IA MSC_24 h group shows a much reduced infarction frequency, particularly surrounding the core as quantized by the Fisher test.

Also, the mean infarct volume of the group treated with IA MSC_24 h group (4.6 mm3, 1.2 to 12.72) was significantly lower as compared to IA PBS_24 h (39.5, 11 to 136.1, p = 0.01) The groups treated with IV_24 h MSCs and IA MSC_24 h did not show a significant reduction in their infarct volume as compared to IA PBS_24 h (Fig. 4A). In order to compare the location of infarcted brain, we used an infarct topography frequency map, which showed a significantly decreased volume of infarction in the penumbral region in the IA MSC_24 h group as compared to the IA PBS_24 h group (Fig 4B).

## Discussion

The major challenge to the IA method of delivery of MSCs is the possibility of vascular occlusion in small arterioles and capillaries owing to the large size of the cells ranging from 15–50 microns [10]. We report a major new finding of a dose response relationship in safety of IA delivery of allogeneic IA MSCs and that lowering of the IA dose overcomes the decrease in blood flow in the middle cerebral artery after IA MSC injection in a rat model of acute ischemic stroke. Furthermore, the lower safe dose of 1×10^5^ MSCs, when given IA at 24 hours results therapeutic effect to reduce infarct volume and improve functional status. Together these findings support further translational investigation to develop intra-arterial allogeneic MSCs as a novel therapy for AIS in the first 24 hours after stroke onset.

With the increasing number of catheter-based endovascular treatments for patients with AIS, intra-arterial delivery of stem cells has tremendous potential for clinical translation. Intra-arterial delivery of bone marrow mononuclear cells into the affected MCA was found to be safe within 3–7 days in a 20 patients trial of patients with MCA strokes [30]. The strategy of endovascular intra-arterial delivery of MSCs has been successfully implemented in early clinical studies of intra-coronary-delivered MSCs after acute myocardial infarction [31], [32]. Given this tremendous promise for clinical application, pre-clinical studies addressing crucial translational hurdles in intra-arterial delivery of stem cells, including MSCs in stroke, are crucial in the efforts to bring this therapy to the bedside.

A previous study of IA MSC delivery reported severely reduced ipsilateral MCA blood flow (80–90%) in 35%, and moderately reduced (10–30%) in 47%, of animals after intra-carotid delivery of 1×10^6^ MSCs given 30 minutes after rMCAo [10]. On dose de-escalation, starting at the dose of 1×10^6^, we found a similar reduction in CBF signal on IA MSC delivery at the doses of 1×10^6^ and 5×10^5^. This adverse effect was somewhat ameliorated at the 2×10^5^dose and resolved with further lowering of the dose to 1×10^5^ consistent with a dose-response relationship in the safety of IA delivery of MSCs in rMCAo. In another recent study the safety of MSC delivery was tested using non-MCAo rats [33]. Lowering of MSCs dose from 2×10^6^ to 1×10^6^ dose decreased stroke lesions on MRI the rodent rMCAo model. Stroke lesions occurred frequently (12 out of 15 animals) when injecting 2×10^6^ MSCs, but not after lowering the dose to 1×10^6^ cells. While the decrease in dose of MCSs led to better safety, similar to our study, the safety of the 1×10^6^ dose is likely due to the use of non-MCAo rats in that study. The twice bigger dose 2×10^6^ produced frequent micro-strokes – mostly in the area of corpus callosum. With regards to mortality post cell injection, the groups receiving 2×10^5^ and higher doses of MSCs had a mortality rate of 42.9% similar to that seen in other studies [10]. The 1×10^5^ dose group and the PBS group had a numerically a lower mortality rate (12.5% and 8.3% respectively) as compared to the higher dose groups although not statistically significant. Similarly, mortality in 5×10^4^ group is numerically higher than 1×10^5^ however, there is no statistically significant difference between two groups. The surgical procedure of MCAo is highly invasive and observed mortality could be confounded by the mortality related to the MCAo in the safety phase of our study.

Despite approximately three times slower speed of injection as compared to a previous study we did not see mitigation of the adverse effect on blood flow in the MCA at the dose of 1×10^6^ MSCs, the only dose tested in that study [10]. The speed of cell injection from our rodent study and others cannot be directly translated in humans. Apart from the large difference in scale, the size of microcatheters available for clinical use allow for excellent blood flow around the microcatheters in the ICA whereas, the PE 10 has a very snug fit in the rodent ICA. Testing of the IA injection speed into ICA in larger animal species may hence be needed.

The reduction of CBF signal over the MCA at the dose of 5×10^5^ is seen shortly after intra-carotid delivery of MSCs at the higher doses and progressively worsens over 60 minutes (Figure 2A), suggesting a mechanical obstruction in the MCA, likely caused by microvascular “sludging” owing to stacking of cells in the microvasculature at higher doses. This is supported by our histology results that showed significantly increased microvascular occlusion at 3–5 days at the higher dose as compared to the lower dose and no evidence for other possible mechanisms for microvascular occlusion such as thrombosis or spasm. The occlusion of microvessels might be due to the higher ratio of the size of some MSCs (some being 15–50 microns in size) to the microvessels size. Along with intravascular MSCs, we found the vast majority of extravascular MSCs to be in the peri-vascular regions at this early time point. We found two separate instances in two separate animal brains of MSCs positioned across the endothelial lining partly inside a capillary and partly in the parenchyma, suggestive of transendothelial migration as the mechanism of transport of these cells into the brain, (Figure 2C, D) as seen in previous studies of intra-carotid stem cell infusion [34].

In the current study we observed the maximum reduction in LDFS was 20% following PBS delivery. Most importantly, the observed reduction in LDFS after PBS delivery occurred shortly after injection and was transient, lasting for short duration (∼5 min) and reverted back to the baseline. In contrast, at the higher doses of 5×10^5^ and 1×10^6^ of IA MSCs, the reduction in LDFS lasted throughout the period of recording (60 min). Hence, the observed transient initial reduction with PBS injection is very unlikely to lead to worsening of ischemic injury

In our study, the animals receiving IA MSCs at the safe dose of 1×10^5^ at 24 hours post rMCAo had a clearly superior functional neurologic outcome at one month as compared to controls receiving IA saline as well as compared to IV MSCs at the same time point. In parallel with the superior functional outcome, we also found the infarct volume to be significantly smaller only in the IA MSC_24 h group as compared to IA PBS_24 h. This decrease in infarct volume was found to be in the penumbral area (Figure 4B) in this group as compared to control suggesting that MSC-mediated neuroprotection as the likely mechanism of benefit. This mechanism has been confirmed in other studies that showed increased concentration of anti-apoptotic factors in the peri-infarct area in animals treated with MSCs.

While previous studies have shown efficacy of IA autologous bone marrow mononuclear cells (BMMCs) and fetal-derived neural stem cells [34], [35], we found that allogeneic MSCs given via the IA route are efficacious in ameliorating neurological deficits in rodent cerebral ischemia without a need for immunosuppression. The application of allogeneic MSCs can facilitate rapid “off-the-shelf” therapy in patients with acute ischemic stroke. We found that not only of 1×10^5^ IA MSCs lead to superior functional neurological recovery as compared to the control group given IA PBS, but also as compared to the group given IV MSCs at a 10-fold higher dose, indicating a robust relative efficacy of the IA route of delivery and its superiority to the IV route when MSCs are administered at 24 hours post rMCAo. Our results are consistent with the functional neurological benefit previously reported [15] with intra-arterial allogeneic MSCs administered at 24 hours post rMCAo. However, the dose of IA MSCs in our study was 10-fold lower as compared to the previous study. Our finding of efficacy at the MTD being safer as compared to higher doses, is novel and supports careful dose escalation studies in clinical translation of IA MSC therapy. In a previous study comparing the IA and IV routes of delivery of autologous BMMCs, efficacy was seen in the IA group but not the IV group [23]. Our results confirm a similar superiority of the IA route but add to this previous finding, as we also found a clear superiority of IA over the IV route on direct comparison of the effect of the two routes of delivery on functional recovery. Of note, in order to compensate for this systemic trapping of IV delivered MSCs, we chose an IV MSC dose that is 10 fold higher than the IA MSC dose so as to serve as an adequate control to IA MSCs that do not undergo systemic trapping. The choice of the IV dose was based on studies showing that IA administration of cells leads to a 7 to 12 times higher number of cells reaching the brain as compared to IV cell administration [34], [36], [37].

The optimal timing of IA stem cell delivery after AIS is unknown. We found that allogeneic IA MSCs given at 24 hours results in efficacy but not when given at 60 minutes after cerebral ischemia. The fact that there was no reduction in the infarct volume in the IA MSC_1 h group indicates that neuroprotection was not significant when MSCs are given at this hyperacute timing after rMCAo. This important finding may be a result of higher concentration of chemoattractant factors for transendothelial transport of MSCs at the more delayed time point, but we did not test this hypothesis. Another study has shown results consistent with the need to delay the administration of IA stem cells after stroke for efficacy [38]. In the prior study of IA neural stem cells in a mouse model of hypoxia-ischemia, authors found that increased phagocytosis of the cells in the post-ischemic brain when transplanted within 24 hours whereas transplantation at 3 days led to lower co-localization of MSCs with pan-monocytic marker Iba-1. These findings suggest that in the hyperacute phase the environment in the post-ischemic brain tissue may be detrimental to the administered IA cells and waiting for this inflammatory response to wane in the first few days is advantageous [38]. Therefore, while administration of IA stem cells via intra-carotid catheters immediately after endovascular reperfusion therapy for ischemic stroke in patients would avoid a second endovascular procedure to administer the cells a few days later, our data suggest that this hyperacute phase may not be an optimal treatment time point for IA cell therapy. Thus early clinical trial designs of IA allogeneic MSCs in ischemic stroke should consider administration of the cells in the first few days after stroke and not in conjunction or immediately following IA thrombolytic therapy.

One of the major limitations of the current study is that we did not test the possibility of slower IA injection to decrease cell sludging. However, the finding of efficacy at the MTD in our study offsets the concern that the higher doses than MTD may be necessary for benefit. Also, we did not comprehensively study the mechanism of action of MSCs, as our primary goal was safety and efficacy of the IA route of MSC delivery. We did not test for time points beyond 24 hours for IA allogeneic MSC injection. Although there is evidence for lack of efficacy of IA BMMC, as beyond 24 hours after rMCAo [39], the outer limit of the treatment time window remains to be established for allogeneic IA MSCs. Additionally, in the present study we monitored the LDF in the MCA for 60 minutes after IA MSCs delivery. However, effect of IA MSCs on LDF beyond 60 minutes is not known and remains to be investigated. Apart from LDF, the information such as presence of MSCs at perivascular locations and overall MSCs survival following IA MSCs needs investigation.

The present study used an experimental model of ischemic stroke in female rats to investigate efficacy of IA MSCs. The MSCs used for IA delivery were generated from male rats. Our approach of delivering MSCs derived from male rats in to the female experimental animals offer an added advantage of localizing MSCs in the brain by cytogenetic approach after IA or IV delivery in future. On the other hand, our use of female rats remains a limitation because it is well known that the circulating ovarian hormones influence ischemic pathology [40]. Future studies employing ovarian hormones-deprived (ovariectomized) female rats are needed to confirm the findings of current study.

In conclusion IA delivery of MSCs at low dose (1×10^5^ cells) did not compromise MCA blood flow in a rat model of MCAo. Importantly, sub-acute delivery of MSCs at lowered dose protected brain from ischemic damage. Our study findings outline the core principles of MSC therapy for ischemic stroke using IA delivery. A clinical translational impact of these results needs to be determined in the future.

## Supporting Information

Table S1 **Physiological variables of protocol I.** Values are mean *±* SD (MABP, mean arterial blood pressure).
(PDF)

Table S2 **Physiological variables of protocol II.** Values are mean ± SD (MABP, mean arterial blood pressure).
(PDF)
