# Supplementary material for: Efficacy and Dose-Dependent Safety of Intra-Arterial Delivery of Mesenchymal Stem Cells in a Rodent Stroke Model
Source: PLoS One. 2014 May 7;9(5):e93735. doi: 10.1371/journal.pone.0093735 (PMC4012944; doi:10.1371/journal.pone.0093735)
Supplement: Table S1 — Physiological variables of protocol I. Values are mean ± SD (MABP, mean arterial blood pressure). (PDF) [file pone.0093735.s001.pdf]

Table 1 Physiological variables of protocol I. Values are mean ± SD (MABP, mean arterial blood pressure)

Group	PBS<br><br>n = 10	$5x10^4$ MSCs<br>n = 7	$1x10^5$ MSCs<br>n = 8	$2x10^5$ MSCs<br>n = 7	$5x10^5$ MSCs<br>n = 7	$1x10^6$ MSCs<br>n = 8
15 min pre-MCAo:						
MABP (mm Hg)	120 ± 19	123 ± 17	128 ± 6	113 ± 24	119 ± 16	125 ± 25
Arterial pH (units)	7.43 ± 0.03	7.43 ± 0.03	7.43 ± 0.03	7.43 ± 0.02	7.42 ± 0.06	7.43 ± 0.04
$P\mathrm{aCO_2}$ (mm Hg)	37 ± 2	38 ± 6	37 ± 4	38 ± 4	38 ± 4	38 ± 2
$P\mathrm{aO_2}$ (mm Hg)	133 ± 43	119 ± 8	145 ± 51	112 ± 19	131 ± 40	127 ± 22
Plasma glucose (mg/dl)	169 ± 33	165 ± 42	155 ± 28	153 ± 22	154 ± 28	183 ± 52
15 min after recirculation:						
MABP (mm Hg)	128 ± 17	116 ± 30	120 ± 12	111 ± 22	131 ± 22	123 ± 12
Arterial pH (units)	7.37 ± 0.05	7.40 ± 0.04	7.39 ± 0.04	7.39 ± 0.01	7.38 ± 0.04	7.39 ± 0.02
$P\mathrm{aCO_2}$ (mm Hg)	41 ± 3	39 ± 7	38 ± 6	40 ± 6	41 ± 3	43 ± 4
$P\mathrm{aO_2}$ (mm Hg)	155 ± 30	129 ± 13	145 ± 26	149 ± 32	129 ± 28	133 ± 18
Plasma glucose (mg/dl)	171 ± 32	168 ± 40	140 ± 26*,&	194 ± 63	199 ± 81	167 ± 35
3 days after MCAo						
Rats' death from brain ischemia (n)	1	4	2	4	3	3

* indicates it is significantly lower than group PBS, $p=0.03$; & indicates that it is lower than $2x10^5$ MSCs group, $p=0.043$ (ANOVA)
