# Supplementary material for: Efficacy and Dose-Dependent Safety of Intra-Arterial Delivery of Mesenchymal Stem Cells in a Rodent Stroke Model
Source: PLoS One. 2014 May 7;9(5):e93735. doi: 10.1371/journal.pone.0093735 (PMC4012944; doi:10.1371/journal.pone.0093735)
Supplement: Table S2 — Physiological variables of protocol II. Values are mean ± SD (MABP, mean arterial blood pressure). (PDF) [file pone.0093735.s002.pdf]

Table 2 Physiological variables of protocol II. Values are mean ± SD (MABP, mean arterial blood pressure)

	1d-IC PBS n = 5	1d-IC MSCs n = 9	1d-IV MSCs n = 8	1h-IC MSCs n = 5
15 min pre-MCAo				
MABP (mm Hg)	128 ± 15	128 ± 14	145 ± 8	123 ± 7
Arterial pH (units)	7.43 ± 0.03	7.43 ± 0.03	7.41 ± 0.06	7.43 ± 0.08
$PaCO_2$ (mm Hg)	37 ± 4	36 ± 2	37 ± 2	33 ± 7
PaO_2 (mm Hg)	125 ± 28	139 ± 38	121 ± 25	154 ± 39
Plasma glucose (mg/dl)	158 ± 14	150 ± 19	151 ± 13	112 ± 35
15 min of recirculation				
MABP (mm Hg)	124 ± 19	139 ± 12	140 ± 13	128 ± 11
Arterial pH (units)	7.37 ± 0.07	7.38 ± 0.03	7.37 ± 0.04	7.37 ± 0.05
$PaCO_2$ (mm Hg)	41 ± 3	39 ± 3	41 ± 4	38 ± 4
PaO_2 (mm Hg)	120 ± 19	141 ± 22	133 ± 36	134 ± 51
Plasma glucose (mg/dl)	184 ± 73	137 ± 21	180 ± 59	167 ± 56
